# Anger Modulates Influence Hierarchies Within and Between Emotional Reactivity and Regulation Networks

**DOI:** 10.3389/fnbeh.2018.00060

**Published:** 2018-04-04

**Authors:** Yael Jacob, Gadi Gilam, Tamar Lin, Gal Raz, Talma Hendler

**Affiliations:** ^1^Sagol School of Neuroscience, Tel Aviv University, Tel Aviv, Israel; ^2^Tel Aviv Center for Brain Functions, Wohl Institute for Advanced Imaging, Tel Aviv Sourasky Medical Center, Tel Aviv, Israel; ^3^The School of Psychological Sciences, Tel Aviv University, Tel Aviv, Israel; ^4^Systems Neuroscience and Pain Laboratory, Department of Anesthesia, Perioperative and Pain Medicine, School of Medicine, Stanford University, Palo Alto, CA, United States; ^5^Film and Television Department, Tel Aviv University, Tel Aviv, Israel; ^6^Sackler Faculty of Medicine, Tel Aviv University, Tel Aviv, Israel

**Keywords:** emotion regulation, fMRI, graph theory network analysis, ventromedial prefrontal cortex

## Abstract

Emotion regulation is hypothesized to be mediated by the interactions between emotional reactivity and regulation networks during the dynamic unfolding of the emotional episode. Yet, it remains unclear how to delineate the effective relationships between these networks. In this study, we examined the aforementioned networks’ information flow hierarchy during viewing of an anger provoking movie excerpt. Anger regulation is particularly essential for averting individuals from aggression and violence, thus improving prosocial behavior. Using subjective ratings of anger intensity we differentiated between low and high anger periods of the film. We then applied the Dependency Network Analysis (D_EP_NA), a newly developed graph theory method to quantify networks’ node importance during the two anger periods. The D_EP_NA analysis revealed that the impact of the ventromedial prefrontal cortex (vmPFC) was higher in the high anger condition, particularly within the regulation network and on the connections between the reactivity and regulation networks. We further showed that higher levels of vmPFC impact on the regulation network were associated with lower subjective anger intensity during the high-anger cinematic period, and lower trait anger levels. Supporting and replicating previous findings, these results emphasize the previously acknowledged central role of vmPFC in modulating negative affect. We further show that the impact of the vmPFC relies on its correlational influence on the connectivity between reactivity and regulation networks. More importantly, the hierarchy network analysis revealed a link between connectivity patterns of the vmPFC and individual differences in anger reactivity and trait, suggesting its potential therapeutic role.

## Introduction

Anger is an omnipresent human phenomenon, with aggression being its prototypical behavioral expression (Averill, 1983; Berkowitz and Harmon-Jones, 2004; Rosell and Siever, 2015). While having an adaptive role in survival, anger may lead to unnecessary violence as well as to personal harmful consequences for health and wellbeing (Johnson, 1990; Chang et al., 2002). Anger regulation is therefore crucial in order to foster prosocial behaviors and avoid negative ramifications for the individual (Davidson et al., 2000; Gilam and Hendler, 2017). Yet despite extensive psychological work on possible regulation strategies and their related processes the neural mechanism that underlies anger regulation is still debated. Unveiling the neural mechanism of anger regulation could serve future diagnosis and therapy in psychiatry, but also improve adaptive prosocial behavior and well-being of individuals prone to anger inducing incidents. In general, emotion regulation refers to a range of strategies humans can voluntarily or involuntarily utilize to modulate emotional experiences, their intensity and expression (Phillips et al., 2008; Gyurak et al., 2011; Etkin et al., 2015). Indeed, humans’ neural circuitry is embedded with networks subserving the regulation of emotional reactivity (e.g., Etkin et al., 2015). To date, it is however, unclear if and how information flow within and between these networks impacts emotion regulation in general and anger regulation in particular.

Recent accounts point to widespread neural activations involved in emotional reactivity and regulation (Ochsner et al., 2012; Etkin et al., 2015). Accordingly, emotional reactivity (e.g., the perception and generation of a threat response) mainly involves subcortical areas, such as the amygdala and periaqueductal gray (PAG), but also cortical regions such as the insula and dorsal anterior cingulate cortex (dACC). Some of these regions are involved in the detection of salient stimuli (Seeley et al., 2007) and others in their rapid evaluation (LeDoux, 1996). Explicit and effortful regulatory processes such as reappraisal (i.e., altering the semantic representation of an emotional stimulus in order to change its affective impact) implicate regions that are typically involved in executive control, such as the dorsolateral PFC (dlPFC)/middle frontal gyrus (MiFG) and superior parietal lobule (SPL), but also inhibition related regions such as the ventrolateral PFC (vlPFC)/inferior frontal gyrus (IFG) and pre-supplementary motor area (pre-SMA). In contrast, implicit and automatic regulation processes (e.g., fear extinction learning in context of a fear conditioning paradigm) has been associated with the ventromedial prefrontal cortex (vmPFC), a major player in self-referential, subjective-valuation and visceromotor control processes (Roy et al., 2012; Hiser and Koenigs, 2018).

Current postulations suggest that successful emotion regulation relies on interactions between the emotion reactivity and regulation systems (e.g., Kober et al., 2008; Lindquist et al., 2012; Etkin et al., 2015). Nevertheless the complex relationships within and between different parts of the proposed systems with regard to the dynamic unfolding of an emotional episode such as anger, remains largely unclear. Indeed, to our knowledge only two studies focused on functional connectivity patterns in regards to anger and its potential regulation. Resting-state fMRI data revealed a positive association between amygdala-orbitofrontal connectivity and the tendency to try to control expressions of anger (Fulwiler et al., 2012). However, this study was limited since it examined only the amygdala’s connectivity with the rest of the brain and did not do so during an actual experience of anger. Moreover, it implemented standard functional connectivity methods that do not address the influencing relationships between brain regions (Hutchison et al., 2013). Effective connectivity methods (Friston, 1994) such as dynamic causal modeling (DCM) and Granger causality analysis (Goebel et al., 2003) were developed to assess such influences. Applying DCM to analyze the brains of individuals watching angry actors demonstrated an increase in the ipsilateral forward connection from the right insula to the right superior temporal gyrus and suppression of the same contralateral connection (Mazzola et al., 2016). Here again participants were not reporting about their anger experience, rather passively observing angry actions of others. Moreover, due to DCM’s inherent limitation, the analysis was conducted on a small subset of regions in the emotion reactivity and regulation networks, precluding a fully comprehensive account of the dynamics within and between these networks.

To examine network organization and hierarchy related to implicit emotion regulation, in this fMRI study participants passively viewed an anger-provoking movie. Previous studies conducted on this dataset have shown that during the viewing of this movie excerpt stronger functional connectivity between the salience network and the limbic medial amygdala network was associated with more intense ratings of emotional experience (Raz et al., 2016), and that activity in participants’ limbic regions was highly synchronized across subjects using inter-subject correlation analysis (Lin et al., 2017).

Here we applied our recent graph-theory based Dependency Network Analysis (D_EP_NA; Jacob et al., 2016) on data acquired while individuals viewed anger-provoking movie excerpts. The D_EP_NA analysis applied computes each brain region’s importance in a given network, according to its effect on the correlations between all other pairs of regions. In this way, the D_EP_NA is able to capture the network’s hierarchy of influence between an *a priori* defined set of regions during different task conditions, and thus to depict the within and between network hierarchies (Jacob et al., 2016; see also Supplementary Material 1 for details on the method). Here we applied D_EP_NA to assess the hierarchy within and between the emotional reactivity and regulation networks, with respect to changes in self-reported anger intensities induced by a political documentary movie excerpt.

**Figure 1:**
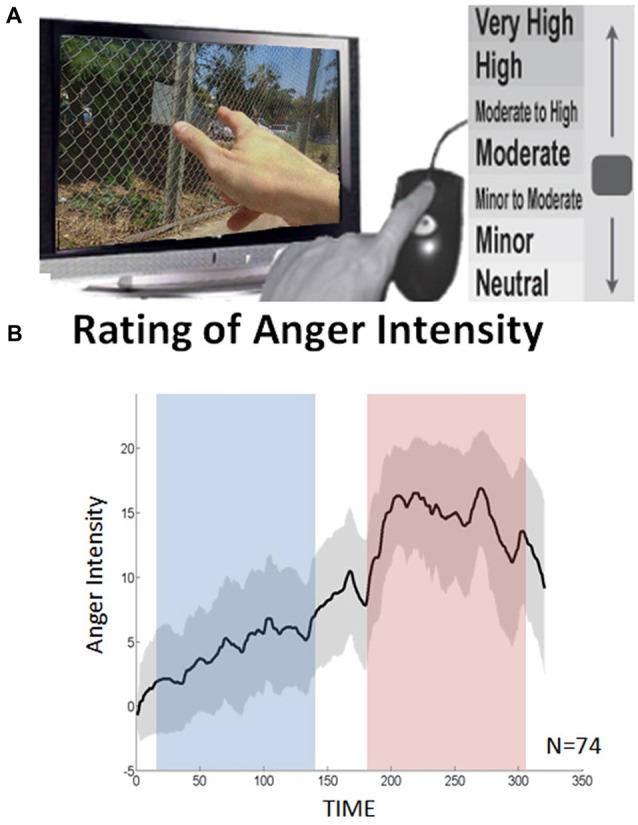
Continuous anger intensity rating.** (A)** The participants retrospectively reported shifts in intensity of anger experienced while watching the clip. **(B)** The averaged continuous emotion rating over all 74 subjects. The dashed lines indicate the standard deviation. Using Wilcoxon signed rank test, a significant difference was found between the low and high anger conditions (blue and red rectangles respectively) in terms of reported anger intensity (*p* < 5 × 10^−14^).

Based on emotional ratings provided by participants following the passive viewing of the movie excerpt in the scanner, D_EP_NA indices were calculated separately during two epochs in the movie defined as high and low anger experience. The anger rating indicated that the selected movie was highly effective in inducing a dynamic anger experience in which anger intensity culminates just before the end of the scene (Figure 1). To note, participants were passively watching the movie excerpt and were not explicitly instructed to apply a specific emotion regulation strategy (as commonly done during explicit emotion regulation paradigms, e.g., Buhle et al., 2014). We therefore assume that implicit emotion regulation was spontaneously applied, designated by the level of emotional experience which unfolded throughout the cinematic scene. This assumption stems from work in psychology showing that there is variability among people in how they rate high intensity stimuli and it has been suggested to be partly related to their regulation tendency (Gross, 1998; Gross and John, 2003).

Based on the previously described idea of dual network involvement and the relation between emotion intensity and tendency for regulation, we therefore hypothesized that the reported increase in anger intensity would involve modulations in connectivity within and between neural networks that are implicated in emotion reactivity (including the insula and the amygdala), and executive control (including IFG and MiFG; Hypothesis 1). Furthermore, the vmPFC which is considered a major player in the implicit emotional regulation network (Etkin et al., 2015) is expected to be particularly influential on network organization during high anger moments (Hypothesis 2). Lastly, difference in network hierarchy between the anger conditions was expected to correlate with both self-reported angry feelings during the movie (i.e., anger state) and anger and emotional regulation tendencies (i.e., anger trait; Hypothesis 3).

## Materials and Methods

### Participants

Valid data were collected from 74 subjects (19.51 ± 1.45 years, males only, plus 27 dropouts) with no known history of neurological or psychiatric disorders and 12 years of education, who volunteered to participate in this study. The study was carried out in accordance with the recommendations of the Declaration of Helsinki and approved by the ethics committee of the Tel Aviv Sourasky Medical Center with written informed consent from all subjects. Part of the dataset was used in previous publications (see Raz et al., 2016; Lin et al., 2017).

### fMRI Data Acquisition

All scans were obtained by a GE 3T Signa Excite echo speed scanner with an 8-channel head coil located at the Wohl Institute for Advanced Imaging at the Tel-Aviv Sourasky Medical Center. Structural scans included a T1-weighted 3D axial spoiled gradient echo (SPGR) pulse sequence (TR/TE = 7.92/2.98 ms, slice thickness = 1 mm, flip angle = 15°, voxel size = 1 mm^3^, FOV = 256 × 256 mm). Functional whole-brain scans were performed in interleaved order with a T2*-weighted gradient echo planar imaging pulse sequence (time repetition (TR)/TE = 3000/35 ms, flip angle = 90°, voxel size = 1.56 × 1.56 × 3 mm^3^, FOV = 200 × 200 mm, slice thickness = 3 mm, 39 slices per volume). Active noise canceling headphones (Optoacoustics) were used.

### fMRI Data Preprocessing

Preprocessing was performed using Brain Voyager QX version 2.4. Head motions were detected and corrected using trilinear and sinc interpolations respectively, applying rigid body transformations with three translation and three rotation parameters. The data were high pass filtered at 0.008 Hz. Spatial smoothing with a 6 mm FWHM kernel was applied. To avoid the confounding effect of fluctuations in the whole-brain BOLD signal, for each TR, each voxel was scaled by the global mean at that time point. Anatomical SPGR data were standardized to 1 × 1 × 1 mm and transformed into Talairach space. SPGR images were then manually co-registered with the corresponding functional maps.

### Anger Inducing Film Excerpt

All subjects underwent fMRI while passively viewing an excerpt from an Israeli documentary film “Avenge But One of My Two Eyes” (Mograbi, 2005), that was previously used in our lab to induce anger (Raz et al., 2016; Lin et al., 2017). The excerpt introduces a fierce political confrontation between the director and soldiers at a checkpoint in the West Bank. The duration of the clip was 5:21 min, and the display was preceded and followed by a 30 s epoch during which the participants passively gazed at an all-black slide.

### Continuous Self-Reporting of State Anger Intensity

A continuous rating of anger intensity was obtained retrospectively (Figure 1A): 70 of the 74 participants watched the movie excerpt for a second time once again outside the scanner and continuously reported on shifts in intensity of anger experienced during the first viewing in the scanner. Retrospective rating was performed over online recording during the fMRI session to avoid the interference of deliberate introspection with the anger experience. The resemblance between the retrospective and online rating was validated in a previous study (Raz et al., 2012). In this study, participants watched the same movie three times. During the second and third viewings, the participants were asked to provide a continuous retrospective report on their emotional experience during the first viewing. The average correlation between these two reports (test-retest reliability) was 0.93. In a complementary test, emotional ratings obtained during first and second (retrospective report) viewings were compared. The correlation between these ratings (construct validity) was 0.89.

The data were acquired via designated in-house software. By using the computer-mouse, subjects indicated changes in their felt intensity of anger in relation to a vertical scale continuously presented on the screen. The scale included seven levels of anger, from neutral to very strong, each containing 3° of change (21° in total). The feedback was sampled at a rate of 10 Hz. The individual overall average intensity was computed by the area under the curve (AUC) of the continuous anger intensity. The higher the AUC the more anger the subject reported over the entire movie excerpt.

Two emotional states of high and low anger were differentiated based on the continuous rating of anger intensity (Figure 1B). Median ratings across all subjects were calculated for all data points. Based on these values, a Matlab script identified one high anger epoch and one low anger epoch. The pair of epochs was defined so that the epochs: (1) were equal in length; (2) significantly differed in median ratings across subjects at *p* < 0.01 in the Wilcoxon signed rank test; and (3) were as long as possible (starting with half of the time points and iterated downwards). This procedure yielded a unique solution of two intervals of 132 s in which the median anger rating across all participants reached its maximum (195–327 s; hereby termed high-anger period), and minimum (36–168 s; hereby termed low-anger period).

### Trait Measure of Anger

The gold standard state-trait anger expression inventory-2 (STAXI-2; Spielberger et al., 1999) was used to assess trait anger, calculated as the sum score in 10 items rated on a 4-point frequency scale from 1 (not at all) to 4 (very much). Subjects are asked to report on the frequency of angry feelings experienced over time. Trait anger measures were assessed from 54 out of the 74 participants.

### Trait Measures of Emotional Regulation

To assess the habitual tendency to use emotion regulation strategies we used the emotion regulation questionnaire (ERQ; Gross and John, 2003). This 10-item questionnaire assesses individual differences in the use of two emotion regulation strategies: reappraisal and suppression. Items are measured on a seven-point Likert scale, from 1 (strongly disagree) to 7 (strongly agree). Emotional regulation traits were assessed in 54 of the 74 participants.

### Emotional Reactivity and Regulation Networks of Interest

The networks of interest were adopted from Etkin et al. (2015). The emotion regulation network consisted of eight regions of interest and the coordinates of these regions were extracted from a meta-analysis on cognitive reappraisal (Buhle et al., 2014). The coordinates for the vmPFC, which was added to the regulation network, and the reactivity network, consisted of six regions extracted from a meta-analysis of neuroimaging studies of emotion (Kober et al., 2008). For details regarding these regions of interest (ROIs) see Table 1. For each ROI we created a spherical mask (radius = 3 mm) centered on the peak x, y, z Talairach coordinates. The averaged BOLD signal (time series) was then extracted for each ROI mask image and each subject.

**Table 1:** High vs. low anger conditions *t*-test results.

Region	High anger (Mean ± std)	Low anger (Mean ± std)	*t* (df)	*p*
**“Influencing degree”**				
L Amy	0.39 ± 0.22	0.35 ± 0.20	0.89 (73)	0.37
R Amy	0.37 ± 0.28	0.34 ± 0.19	1.03 (73)	0.31
L midIns	0.46 ± 0.36	0.38 ± 0.24	1.39 (73)	0.17
R midIns	0.39 ± 0.30	0.29 ± 0.17	2.40 (73)	0.02*
PAG	0.29 ± 0.30	0.26 ± 0.19	0.53 (73)	0.60
dACC	0.42 ± 0.28	0.41 ± 0.25	0.49 (73)	0.63
L IFG	0.45 ± 0.30	0.40 ± 0.27	1.17 (73)	0.25
R IFG	0.34 ± 0.25	0.31 ± 0.20	0.36 (73)	0.72
L MiFG	0.33 ± 0.27	0.32 ± 0.22	0.66 (73)	0.51
R MiFG	0.29 ± 0.15	0.29 ± 0.17	0.22 (73)	0.83
L SPL	0.33 ± 0.18	0.34 ± 0.23	−0.26 (73)	0.80
R SPL	0.30 ± 0.25	0.27 ± 0.15	0.62 (73)	0.53
preSMA	0.35 ± 0.20	0.35 ± 0.22	0.42 (73)	0.68
vmPFC	0.32 ± 0.22	0.24 ± 0.15	3.11 (73)	0.0026**
**Intra-network influence**				
L IFG	0.26 ± 0.21	0.24 ± 0.25	0.50 (73)	0.62
R IFG	0.17 ± 0.13	0.16 ± 0.12	0.52 (73)	0.60
L MiFG	0.22 ± 0.18	0.21 ± 0.19	0.35 (73)	0.72
R MiFG	0.22 ± 0.16	0.21 ± 0.15	0.36 (73)	0.72
L SPL	0.24 ± 0.16	0.26 ± 0.21	−0.71 (73)	0.48
R SPL	0.17 ± 0.16	0.18 ± 0.16	−0.41 (73)	0.68
preSMA	0.21 ± 0.14	0.21 ± 0.16	−0.02 (73)	0.99
vmPFC	0.19 ± 0.15	0.14 ± 0.12	2.90 (73)	0.005**
**Influence on connections within the reactivity network**				
L IFG	1.43 ± 1.56	1.24 ± 0.89	0.97 (73)	0.34
R IFG	0.92 ± 1.11	0.89 ± 0.91	0.18 (73)	0.86
L MiFG	0.77 ± 1.42	0.63 ± 0.64	0.83 (73)	0.41
R MiFG	0.49 ± 0.44	0.51 ± 0.47	−0.29 (73)	0.78
L SPL	0.59 ± 0.54	0.54 ± 0.48	0.60 (73)	0.55
R SPL	0.66 ± 1.07	0.52 ± 0.46	0.95 (73)	0.35
preSMA	0.72 ± 0.88	0.61 ± 0.50	0.93 (73)	0.36
vmPFC	0.65 ± 0.62	0.63 ± 0.72	0.26 (73)	0.79
**Influence on connections between the regulation and reactivity networks**				
L IFG	0.032 ± 0.023	0.029 ± 0.019	1.20 (73)	0.23
R IFG	0.026 ± 0.020	0.024 ± 0.016	0.35 (73)	0.73
L MiFG	0.023 ± 0.020	0.021 ± 0.016	0.61 (73)	0.54
R MiFG	0.019 ± 0.011	0.019 ± 0.013	0.20 (73)	0.84
L SPL	0.023 ± 0.013	0.023 ± 0.015	−0.28 (73)	0.78
R SPL	0.022 ± 0.018	0.020 ± 0.011	1.02 (73)	0.31
preSMA	0.026 ± 0.015	0.025 ± 0.017	0.26 (73)	0.80
vmPFC	0.024 ± 0.017	0.018 ± 0.011	3.03 (73)	0.0033**

*p < 0.05, **q < 0.05, FDR corrected.

### Dependency Network Analysis (D_EP_NA)

**Figure 2:**
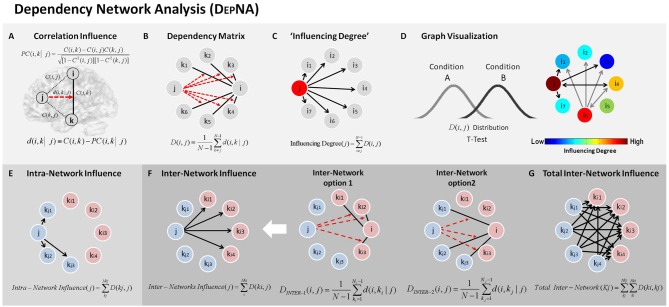
Dependency network analysis (D_EP_NA). Step 1:** (A)** Partial correlation coefficient—a statistical measure indicating how a third variable affects the correlation between two other variables. For example the partial correlation between nodes *i* and *k* with respect to a third node *j*—PC(*i*, *k*| *j*) defined in the equation. Where C(*i*, *k*), C(*i*, *j*) and C(*k*, *j*) are the regions of interest (ROI)-ROI correlations. We then define the influence of *j* on the pair of elements *i* and *k* as the difference between the correlation and the partial correlation. Step 2: **(B)** Dependency Matrix—Next, we calculate the partial correlation effect for each ROI on all other pairwise correlations in the network. We define the total influence of node *j* on node *i*, D(*i*, *j*) as the average influence of node *j* on the correlations C(*i*, *k*), over all nodes *k*. The node dependencies define a dependency matrix D, whose (*i*, *j*) element is the influence of node *j* on node *i*. Step 3:** (C)** “Influencing Degree”—We then define the influences of node *j* as the sum of the influence D(*i*, *j*) of *j* on all other nodes *i*. The higher this measure the more this node influenced all other connections in the network. Step 4: **(D)** Graph Visualization –Each ROI is color-coded according to its influencing or influenced degrees. All pairwise ROIs with dependency elements D that are significantly different between two conditions (or groups) at the *p* < 0.05 level are plotted as edges. Each edge is color-coded according to the *t*-test sign as light or dark gray. The arrows represent the direction of influence. **(E)** Intra-Network influence—The influence within the sub-network is computed for each node as the sum of its influences on the nodes within its network. **(F)** Inter-Network influence—The influences between the sub-networks is calculated for two different options: (1) as the sum of the influences of a node from one network only on the connections within the second network; and (2) as the sum of the influences of a node from one network only on the connections between the first (*kj*) and the second (*ki*) networks. **(G)** Total inter-network—total influences between the sub-networks were computed as the sum of all inter-network influences from one network on the nodes within the second network.

During each of the low and high movie periods we applied the D_EP_NA method to probe relationships of influence between the network nodes. The D_EP_NA and its implementation to fMRI was previously described extensively (Jacob et al., 2016). The D_EP_NA steps needed to calculate the networks’ ROIs influence are described in Figure 2. Further details on the D_EP_NA features, characteristics and interpretations are described in Supplementary Table S1 in the Supplementary Material 1.

Briefly, the ROI-ROI correlations were calculated by Pearson’s formula (Rodgers and Nicewander, 1988). First we normalized the correlation coefficients by using a Fisher Z transformation. Next, we used the resulting normalized ROI correlations to compute partial correlations (Baba, 2004; Figure 2A). The partial correlation coefficient is a statistical measure indicating how a third variable affects the correlation between two other variables (Shapira et al., 2009). The partial correlation between nodes *i* and *k* with respect to a third node *j*—*PC (i,k|j)* is defined as:

(1) $$PC\left(i,k|j\right)=\frac{C(i,k)-C(i,j)C(k,j)}{\sqrt{\left[1-C^{2}(i,j)][1-C^{2}(k,j)\right]}}$$

Where *C(i,j)*, *C(i,k)* and *C(k,j)* are the ROI-ROI correlations. The relative effect of the correlations *C(i,j)* and *C(k,j)* of node *j* on the correlation *C(i,k)*  (Kenett et al., 2010; Figure 2A), is given by:

(2) d(i,k|j)≡C(i,k)−PC(i,k|j)

This quantity is large only when a significant fraction of the correlation between nodes *i* and *k* can be explained in terms of node *j*. To avoid cases where we sum over positive and negative influences, we reset all negative values to zero.

We then define the total influence of node *j* on node *i*, or the dependency *D(i,j)* of node *i* on node *j* to be (Figure 2B):

(3) $$D(i,j)=\frac{1}{N-1}\sum_{k\neq j}^{N-1}d(i,k|j)$$

As defined, *D(i,j)* is a measure of the average influence of node *j* on the correlations *C(i,k)*, over all nodes *k*. *N* is the number of nodes in the network. The node activity dependencies define a dependency matrix *D* whose *(i,j)* element is the influence of node *j* on node *i*.

Next we sorted the nodes according to the system level influence of each node on the correlations between all other node pairs (Figure 2C). The system level *“Influencing Degree”* of node *j* is simply defined as the sum of the influence of node *j* on all other nodes *i*, that is:

(4) $$Influencing\;Degree\;(j)=\sum_{1\neq j}^{N-1}D(i,j)$$

The D_EP_NA *“Influencing Degree”* measure indicates the hierarchy of efferent (out-degree) impact of the node on the entire network (or sub-network). The higher this measure is, the greater its impact on all other connections in the network and the more likely it is to be generating information flow in the network.

To create network graph visualization we used the pairwise dependency connectivity matrix. A two-tailed *t* statistic was computed to compare the two conditions (e.g., high vs. low anger epochs). We then connect only pairwise ROIs with dependencies that were significantly different between the two conditions (*p* < 0.05 level) creating a simple graph visualization of the differences between the conditions across all subjects. The brain visualization of the graph was conducted with the BrainNet Viewer (Xia et al., 2013)^1^.

The D_EP_NA was computed for each subject for each period (i.e., high- and low-anger) resulting in an *“Influencing Degree”* for each region (Figure 2C). We then conducted a between-periods paired *t*-test for each region’s *“Influencing Degree”* (total of 14 ROIs). The results were corrected for multiple comparisons using FDR (Benjamini and Hochberg, 1995) correcting for 14 tests.

In addition, we further investigated the intra and inter-network influence hierarchies for the reactivity and regulation networks as two separate sub-networks. The intra-network influence was computed for each network node’s influence on the connections within its network (Figure 2E). We then conducted a between-periods paired *t*-test for each of the regulation network regions’ (eight ROIs) intra-network influence degree. The results were corrected for eight multiple comparisons using FDR correction.

The inter-network influences were divided into two cases: (1) the sum of the influences of a node from one sub-network only on connections within the second sub-network (i.e., inter-influence 1, Figure 2F); and (2) the sum of the influences of a node from one sub-network only on connections between the two sub-networks (i.e., inter-influence 2, Figure 2F). Finally, the total network influence on the second network was calculated as the sum of the inter-network influences. This feature was calculated for each inter-network influence option separately. Next, for each network configuration (i.e., inter influence 1 and 2), we conducted a between-periods paired *t*-test for each region’s inter-influence degrees (total of 14 ROIs). The results were corrected for 14 multiple comparisons tests using FDR correction.

Hypothesis 1 was tested by the total inter-network influence of the regulation network on the reactivity network. Hypothesis 2 was tested by the nodal influence on several network levels: (1) influence on the entire two networks’ brain regions; (2) intra-network influence within the regulation network (eight ROIs); (3) inter-network influence of the regulation network regions on the connections within the reactivity network; and (4) inter-network influence of the regulation network regions on the connections between the reactivity network and the regulation network. Hypothesis 3 was tested by correlating tests 1–4 from hypothesis 2 with anger measures (anger intensity and trait anger) and trait emotional regulation (reappraisal and suppression) correcting for the two hypotheses within each test using FDR. Pearson correlations were performed to assess the association between the results that were found to be significantly different between the high and low anger epochs (i.e., low-anger minus high-anger “Influencing Degree”) and subjects’ anger or emotional regulation measures. Subjects whose values exceeded the mean by three standard deviations were excluded from the analysis.

### Estimating the Spatial Specificity of the Results

In order to examine whether our D_EP_NA findings are specific to the networks and to control for whole brain effects of physiological parameters such as respiration, heartbeat, or head motion, we performed a bootstrapping procedure. The observed *t* and *r* values resulting from the previous analyses were compared with corresponding values generated by identical analyses of random sets of gray matter regions. The D_EP_NA findings were obtained using different network configurations with 8 or 14 regions. Accordingly, depending on the relevant configuration, the randomized networks contained 8 or 14 regions. The coordinates of these regions were randomly selected from a sampling space, defined based on ICBM 452 probability map^2^. The mask was created by thresholding ICBM 452 map to exclude voxels with probability lower than 50% of being classified as gray matter.

We performed a *post hoc* assessment of the specificity of the D_EP_NA paired *t*-test findings by comparing the observed *t*-value with the results of the randomized networks. The *t-value* background distribution was generated by repeating this procedure 1000 times. The *p* value of the bootstrapping test was defined as the fraction of the number of random cases which obtained t statistic values smaller than the observed finding, given as

(5) $$p=\frac{\sum Random\;Nettvals\leq Original\;t\;vals)+1}{k+1}$$

where *k* is the number of random tests (*k* = 1000).

Since the observed correlation to behavior effect was tested only on a single network node, one region was randomly selected from each of the 1000 random networks and its D_EP_NA measure was correlated to the individual behavioral indices. This procedure was repeated for each behavioral index separately (i.e., anger intensity, trait anger and trait emotional regulation), resulting in a background distribution of 1000 correlation coefficients.

The *p* value of the random correlations test was defined as the fraction of the number of random regions which obtained higher/lower correlation coefficient *r* values than the observed findings.

## Results

### Within and Between Networks’ Hierarchy With Respect to Anger

To test our first hypothesis and evaluate if the total influence of the regulation network on the reactivity network was higher during the high anger epoch compared to the low anger epoch, we calculated the D_EP_NA total inter-network influence measures of all the regulation network regions. At odds with our expectations, we found that these measures were not significantly different between the low vs. high anger epochs both in the inter-network influence analysis on the connections within the reactivity network (*p* > 0.1), and for the inter-network influence analysis on the connections between the reactivity and regulation networks (*p* > 0.2).

To test our second hypothesis about whether the implicit regulation related region (i.e., the vmPFC) exhibited higher influence during the high anger epoch, we applied the D_EP_NA influencing index on three different network configurations: (1) the entire set of regions (i.e., emotional reactivity and regulation networks), by calculating the *“Influencing Degree”* measure; (2) the within emotion regulation network influence, by calculating the “Intra-network Influence” measure; and (3) the emotional regulation influence on the emotional reactivity network, by calculating the “Inter-Networks Influence” measure.

**Figure 3:**
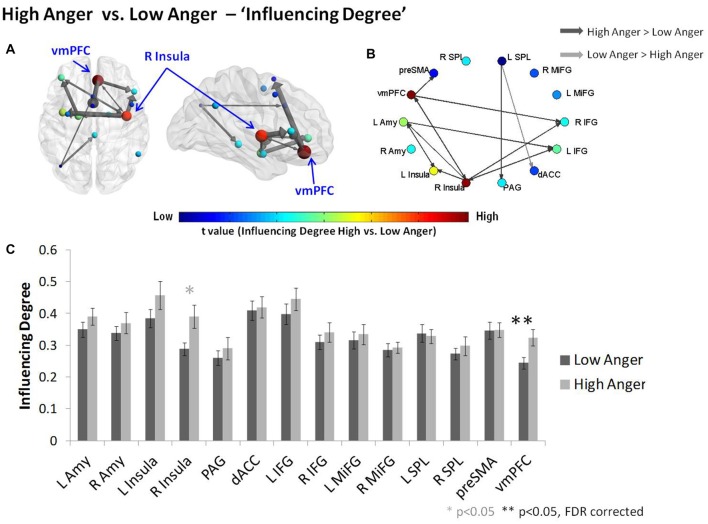
Characterizing anger states by D_EP_NA “Influencing Degree”. The “Influencing Degree” network graph constructed for high anger minus low anger. The reactivity-regulation network illustrated on sagittal and axial views **(A)** and graphs visualization **(B)**. Each ROI was sized and color-coded according to its “Influencing Degree”. Warmer colors and bigger sphere represent a more influencing node. All pairwise ROIs with connections that were significant at the *p* < 0.05 level, using a *t*-test, are plotted as edges. The arrows indicate the direction of the influence, and the arrows’ size represents the t-statistic. **(C)** The nodes’ “Influencing Degree” averaged over all 74 subjects. The influence of the ventromedial prefrontal cortex (vmPFC) was significantly higher in the high anger condition vs. low anger.

The difference in each node’s *“Influencing Degree”* (low vs. high anger; Figure 3 and Table 1) indicated the vmPFC (*t* = 3.11, *p* < 0.003, *q* < 0.05 FDR corrected) and right insula (*t* = 2.4, *p* < 0.02) had higher influence on both reactivity and control networks in the high anger condition compared to low anger (Figure 3C). We note that the right insula result did not withstand correction for multiple comparisons, therefore, we consider it only as a trend. The vmPFC t value result was also found to be significant (*p* < 0.05) using a control permutation test conducted on 1000 random networks each consisting of 14 random regions (see Supplementary Figure S1A in Supplementary Material 1). The graph visualization showed that the vmPFC had higher influence specifically on the right Insula (*t* = 2.30, *p* < 0.03), right IFG (*t* = 2.62, *p* < 0.02), left MiFG (*t* = 2.31, *p* < 0.03), left SPL (*t* = 2.06, *p* < 0.05) and pre-SMA (*t* = 3.32, *p* < 0.002; Figure 3B).

**Figure 4:**
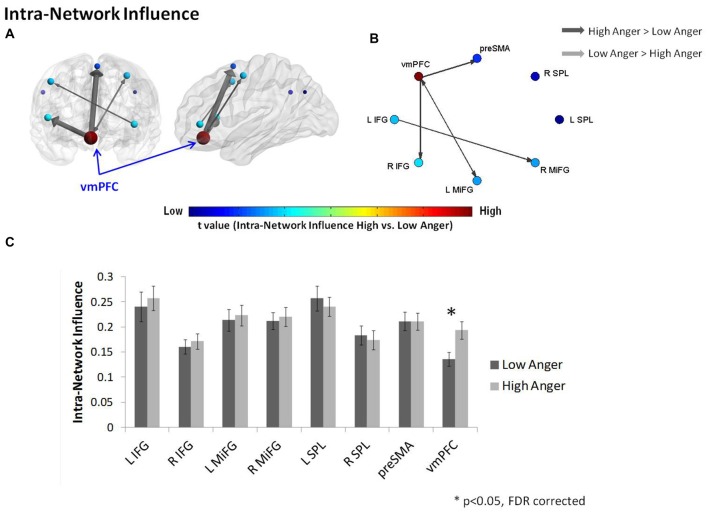
Intra-regulation network results. The intra-network graph constructed for high anger minus low anger. The regulation network illustrated on sagittal and coronal views **(A)** and graphs visualization **(B)**. Each ROI was sized and color-coded according to its intra-network influence degree. Warmer colors and bigger sphere represent a more influential node. All pairwise ROIs with connections that were significant at the *p* < 0.05 level, using a *t*-test, are plotted as edges. The arrows indicate the direction of the influence, and the arrow size represents the *t*-statistic. **(C)** The nodes’ intra-network influence degree averaged over all 74 subjects. The vmPFC influence within the regulation network was significantly higher in the high anger condition vs. low anger.

In order to further investigate the vmPFC’s specific impact on the connections within the regulation network, we conducted an intra-network influence analysis (see Figure 2E). The vmPFC exhibited higher influence on the regulation network during the high anger epoch (*t* = 2.9, *p* < 0.005, *q* < 0.05 FDR corrected; Figure 4). The spatial specificity of this result was *p* < 0.05 (see Supplementary Figure S1B in Supplementary Material 1).

**Figure 5:**
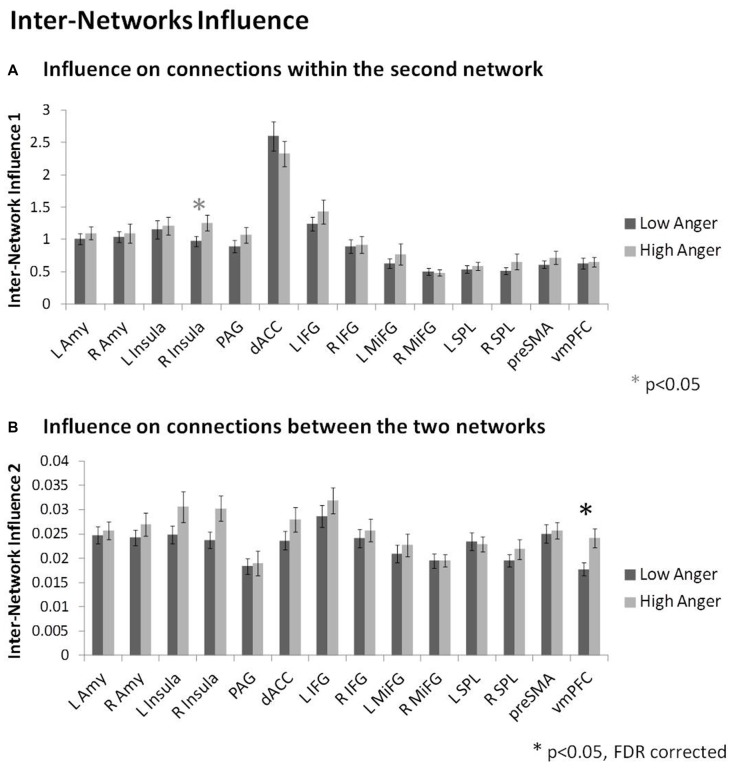
Inter-Network network results. The nodes’ inter-network influencing degree averaged over all 74 subjects for the influence on connections within the second network **(A)**, and the influence on connections between the two networks **(B)**. The vmPFC influence on the connections between the reactivity network and the regulation network was significantly higher in the high anger condition vs. low anger.

In order to specifically investigate communication between the regulation and reactivity networks we conducted the D_EP_NA inter-network analysis. Analysis of the influence of a region on the connections within the second network (i.e., inter-network option 1, see Figure 2F) found that the right insula had higher influence on the connections within the regulation network regions (*t* = 2.01, *p* < 0.05) in the high anger compared to the low anger condition (Figure 5A). We note that this result did not withstand correction for multiple comparisons, therefore, we consider it only as a trend. Analysis of the influence of a region on the connections between the two networks (i.e., inter-network option 2, see Figure 2F) found that the vmPFC had significantly higher influence on the connections between the reactivity and regulation network regions (*t* = 3.03, *p* < 0.003) in the high anger compared to the low anger condition (Figure 5B). The spatial specificity of this result was *p* < 0.05 (see Supplementary Figure S1C in Supplementary Material 1).

### The Relation Between Individual Anger Related Networks’ Hierarchy and Behavior

**Figure 6:**
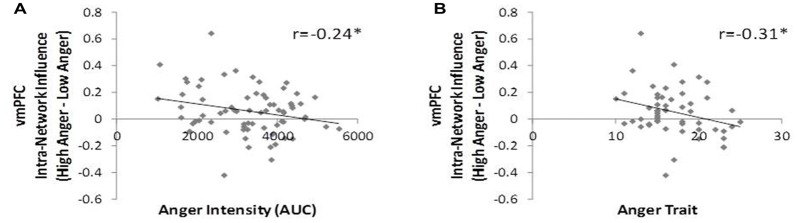
D_EP_NA features association to behavior. The vmPFC intra-network influence was found in negative correlation with the anger intensity measure during the movie excerpt **(A)** and with trait-anger measure **(B)**. Therefore, the more the subject reported higher anger intensity and the more they tend to get angry in general, the lower was the vmPFC influence on the rest of the regulation network regions. *p < 0.05, FDR corrected.

To test our third hypothesis regarding the relation between networks’ hierarchy and behavioral indications of anger state and tendencies we investigated the correlation between the level of vmPFC influence indices and the level of anger intensity, trait anger and trait emotional regulation. The vmPFC intra-network influence negatively correlated with anger intensity during the movie excerpt (*r* = −0.24, *p* < 0.04, *n* = 70, Figure 6A) and with trait anger (*r* = −0.31, *p* < 0.03, *n* = 51, Figure 6B). The spatial specificity of these results was *p* < 0.003 (see Supplementary Figure S2 in Supplementary Material 1). In other words, higher subjective reported anger intensity and higher tendency to become angry were associated with lower vmPFC influence on regions of the regulation network.

## Discussion

The goal of this study was to utilize the D_EP_NA to investigate the properties of the hierarchy between the emotion reactivity and regulation networks during an anger experience induced by a movie excerpt. Applying the D_EP_NA during high- and low anger states depicted by individual self- report, revealed related modifications in network hierarchy. Specifically, D_EP_NA identified the vmPFC regulation-related region as a central node during the high anger episode based on its impact both on whole system connectivity, and on intra- and inter network connectivity. We demonstrated that lower vmPFC influence within the regulation network was associated with higher anger intensity reported during anger induction, and higher trait anger, emphasizing its relation to the direct experience of induced anger and to the habitual tendency to be angry. Finally, we demonstrated that higher vmPFC impact on inter-network connections was associated with a higher tendency to apply a suppression regulation strategy, linking vmPFC’s inter-network influence to an independent measure assessing the engagement of implicit emotion regulation strategies. Together these results support and replicate our previous findings in which vmPFC played a key role in spontaneous anger regulation during an interpersonal induction of anger, as well as correlating with trait suppression (Gilam et al., 2015), and extend them by demonstrating a unique directional connectivity pattern with regulation and emotional reactivity related networks. Of note however, this study was limited by being conducted on relatively young male participants (19.51 ± 1.45 years), thus it may not reflect the general population. More so, anger was provoked using only one film excerpt and was not compared to other emotional experiences, thus precluding specific claims on anger processing *per se*.

As might be expected from previous imaging work on implicit emotion regulation (Etkin et al., 2006), the regulation network as a whole, did not demonstrate higher total influence on the reactivity network during high- compared to low- anger epochs as tested by the total inter-network influence analysis. One possible explanation for this result is the fact that in this study subjects were not specifically instructed to regulate their emotions. The only brain region within the regulation network related to implicit emotional regulation processes is the vmPFC. Examining the total influence may have masked more subtle vmPFC influences related to implicit regulation effects. In line with this notion and confirming our second hypothesis, comparison of each network region’s *“Influencing Degree”* between the high- and low-anger conditions revealed that the influence of the vmPFC was increased in the high anger condition (Figure 3, Table 1). The vmPFC also exhibited higher intra-network influence within the regulation sub-network during the high anger epoch (Figure 4C). This may indicate a central role of vmPFC during the experience of anger and potentially the inherent involvement of regulatory processes in such an experience (Gilam and Hendler, 2017). The nature of our methodology is important to emphasize. Rather than standard BOLD (co)activation analyses which provide information regarding the general involvement of various brain regions during the emotional experience, D_EP_NA provides us with a clue regarding the brain regions that drive the emotional regulation process. The significant change in vmPFC impact on regions of the reactivity and regulation network may reflect its involvement in a process that aims to exert control over the anger that stems from the provocation. This may be supported by the negative relationship between the level of vmPFC influence on regulation regions and the level of reported anger intensity (Figure 6A). However, since participants were not directly instructed to regulate their emotions, we cannot know for sure whether this relation is indeed due to greater emotion regulation while viewing the anger-emotive film.

Indeed the vmPFC has been indicated in several other affect related processes such as reward valuation processes (Levy and Glimcher, 2012) and social information processing (Bechara et al., 2000; Rolls, 2004; Adolphs, 2009; Mitchell, 2009).

The broad network perspective as captured by the D_EP_NA method here, extends our knowledge regarding emotional regulation far beyond anger. It demonstrates that influence hierarchies within and between reactivity and regulation networks rather than activity or co-activation may reflect emotional state modification, even at the individual level. This pattern of results thus challenges the common concept of emotion regulation as a one way down regulation effect of a control network over a reactivity network region (Buhle et al., 2014; Morawetz et al., 2017). Our findings attribute a more active role of the vmPFC in shaping the regulation network organization as well as in its relation with the reactivity network in support of effective emotion regulation. Network hierarchy is one of probably several other metrics that might capture dynamics in network organization with respect to changes in emotional experience. Further studies should test different network features which have been shown to be sensitive to emotional brain states such as network integration (Kinnison et al., 2012) or modularity (Ben Simon et al., 2017) during the anger experience. It should also be noted that while D_EP_NA can be used to make inferences regarding the influence hierarchy within a network, it does not infer a causal influence in the true sense, as correlation does not imply causation (Friston, 2009). We therefore suggest that the D_EP_NA results may target the critical regions to delineate the specific model of connectivity required for causality testing methods such as the DCM. Our results thus indicate that further studies using DCM should test the causality between the vmPFC, right IFG and right Insula (see Figure 3B).

One caveat that should be mentioned here is the inherent difficulty of making decisive scientific interpretations when using complex and naturalistic experimental stimuli such as movies. Given the fact that the brain has evolved to cope with/to process a continuous flux of multisensory input, naturalistic experiments may provide important insights into processes that are not optimally captured by classically controlled studies (Adolphs et al., 2016; Gilam and Hendler, 2016), however they are also prone to misinterpretation due to confounding variables. In our specific case, the low- and high anger phases of the movie possibly differ in audiovisual parameters that potentially contribute to the emotional intensity, but may not be considered as emotional *per se* (e.g., loudness and optical flow). In this case, our findings on differences in influence between the phases may be related to perceptual features rather than emotional factors. However, this caveat is addressed in our analysis of the relations between the influence levels on the one hand, and the reported acute anger intensity and trait anger on the other. In these cases, the variance of the dependent variable (influence level) is explained by independent parameters that are clearly related to emotion. By correlating the influence levels with these emotion measures, we examined their effect on the neural data while keeping the cinematic stimulus constant across subjects (see Raz et al., 2013, for a description of this rationale). An additional claim can be that since in our study the low anger period precedes the high anger period, differences could be attributed to order effects. However, *post hoc* D_EP_NA analyses of two low- and two high subsequent time frames of anger periods do not support such a concern (see Supplementary Figure S3 in Supplementary Material 1). Nevertheless, future controlled experimental designs could potentially contribute to understanding our reported findings on the overall difference of vmPFC influence levels between the more and less emotional epochs in light of low-level changes not related to the induced emotion.

To conclude, using the D_EP_NA approach we demonstrated that high- and low anger states can be characterized by different graph hierarchy of the reactivity and regulation networks. In particular the DepNA metrics depicted the central influence of the vmPFC; a major implicit regulation related node on these networks during the high anger experience. Our findings add to existing research on emotional regulation in general and anger regulation in particular by inspecting the operation and organization of relatively large-scale brain networks, while considering individual differences in state and trait. Specifically, higher anger intensity and trait-anger scores were associated with less vmPFC influence on the regulation network. Assuming the anger experience as provoked by the movie clip indeed initiated an implicit anger regulation process, we suggest that adaptive anger regulation could involve higher vmPFC influence on the regulation network. While anatomical and lesion studies have long indicated the causal role of the vmPFC and its interactions with limbic structures in emotion regulation (e.g., McDonald et al., 1996; Barrash et al., 2000), the system level influence degree measure may comprise a more dynamic fMRI index of this process. We hope that such a measure could be utilized as a target for future neuromodulation therapies aimed to alleviate the negative implications of anger on people’s lives.

## Author Contributions

YJ analyzed the data and wrote the manuscript. TL and GG acquired all data. GR, GG and TH helped interpret the results and wrote the manuscript. TH coordinated and directed the project. All authors reviewed the manuscript.

## Conflict of Interest Statement

The authors declare that the research was conducted in the absence of any commercial or financial relationships that could be construed as a potential conflict of interest.

## Footnotes

^1^http://www.nitrc.org/projects/bnv/

^2^http://www.loni.usc.edu/atlases

## References

[B1] Adolphs R. (2009). The social brain: neural basis of social knowledge. Annu. Rev. Psychol. 60, 693–716. 10.1146/annurev.psych.60.110707.163514 18771388 PMC2588649

[B2] Adolphs R. Nummenmaa L. Todorov A. Haxby J. V. (2016). Data-driven approaches in the investigation of social perception. Philos. Trans. R. Soc. Lond. B Biol. Sci. 371:20150367. 10.1098/rstb.2015.0367 27069045 PMC4843606

[B3] Averill J. R. (1983). Studies on anger and aggression: implications for theories of emotion. Am. psychol. 38, 1145–1160. 10.1037/0003-066x.38.11.1145 6650969

[B4] Baba K. (2004). Partial correlation and conditional correlation as measures of conditional independence. Aust. N Z J. Stat. 46, 657–664. 10.1111/j.1467-842x.2004.00360.x

[B5] Barrash J. Tranel D. Anderson S. W. (2000). Acquired personality disturbances associated with bilateral damage to the ventromedial prefrontal region. Dev. Neuropsychol. 18, 355–381. 10.1207/s1532694205barrash 11385830

[B6] Bechara A. Damasio H. Damasio A. R. (2000). Emotion, decision making and the orbitofrontal cortex. Cereb. Cortex 10, 295–307. 10.1093/cercor/10.3.295 10731224

[B7] Ben Simon E. Maron-Katz A. Lahav N. Shamir R. Hendler T. (2017). Tired and misconnected: a breakdown of brain modularity following sleep deprivation. Hum. Brain Mapp. 38, 3300–3314. 10.1002/hbm.23596 28370703 PMC6866787

[B8] Benjamini Y. Hochberg Y. (1995). Controlling the false discovery rate: a practical and powerful approach to multiple testing. J. R. Stat. Soc. B 57, 289–300.

[B9] Berkowitz L. Harmon-Jones E. (2004). Toward an understanding of the determinants of anger. Emotion 4, 107–130. 10.1037/1528-3542.4.2.107 15222847

[B10] Buhle J. T. Silvers J. A. Wager T. D. Lopez R. Onyemekwu C. Kober H.. (2014). Cognitive reappraisal of emotion: a meta-analysis of human neuroimaging studies. Cereb. Cortex 24, 2981–2990. 10.1093/cercor/bht154 23765157 PMC4193464

[B11] Chang P. P. Ford D. E. Meoni L. A. Wang N.-Y. Klag M. J. (2002). Anger in young men and subsequent premature cardiovascular disease: the precursors study. Arch. Intern. Med. 162, 901–906. 10.1001/archinte.162.8.901 11966341

[B12] Davidson R. J. Putnam K. M. Larson C. L. (2000). Dysfunction in the neural circuitry of emotion regulation—a possible prelude to violence. Science 289, 591–594. 10.1126/science.289.5479.591 10915615

[B13] Etkin A. Büchel C. Gross J. J. (2015). The neural bases of emotion regulation. Nat. Rev. Neurosci. 16, 693–700. 10.1038/nrn4044 26481098

[B14] Etkin A. Egner T. Peraza D. M. Kandel E. R. Hirsch J. (2006). Resolving emotional conflict: a role for the rostral anterior cingulate cortex in modulating activity in the amygdala. Neuron 51, 871–882. 10.1016/j.neuron.2006.12.003 16982430

[B16] Friston K. J. (1994). Functional and effective connectivity in neuroimaging: a synthesis. Hum. Brain Mapp. 2, 56–78. 10.1002/hbm.460020107

[B15] Friston K. J. (2009). Causal modelling and brain connectivity in functional magnetic resonance imaging. PLoS Biol. 7:e1000033. 10.1371/journal.pbio.1000033 19226186 PMC2642881

[B17] Fulwiler C. E. King J. A. Zhang N. (2012). Amygdala-orbitofrontal resting state functional connectivity is associated with trait anger. Neuroreport 23, 606–610. 10.1097/WNR.0b013e3283551cfc 22617448 PMC4271793

[B104] Gilam G. Hendler T. (2016). With love, from me to you: embedding social interactions in affective neuroscience. Neurosci. Biobehav. Rev. 68, 590–601. 10.1016/j.neubiorev.2016.06.027 27339690

[B18] Gilam G. Hendler T. (2017). Deconstructing anger in the human brain. Curr. Top. Behav. Neurosci. 30, 257–273. 10.1007/7854_2015_408 26695163

[B19] Gilam G. Lin T. Raz G. Azrielant S. Fruchter E. Ariely D.. (2015). Neural substrates underlying the tendency to accept anger-infused ultimatum offers during dynamic social interactions. Neuroimage 120, 400–411. 10.1016/j.neuroimage.2015.07.003 26166623

[B20] Goebel R. Roebroeck A. Kim D.-S. Formisano E. (2003). Investigating directed cortical interactions in time-resolved fMRI data using vector autoregressive modeling and Granger causality mapping. Magn. Reson. Imaging 21, 1251–1261. 10.1016/j.mri.2003.08.026 14725933

[B21] Gross J. J. (1998). Antecedent- and response-focused emotion regulation: divergent consequences for experience, expression, and physiology. J. Pers. Soc. Psychol. 74, 224–237. 10.1037/0022-3514.74.1.224 9457784

[B22] Gross J. J. John O. P. (2003). Individual differences in two emotion regulation processes: implications for affect, relationships, and well-being. J. Pers. Soc. Psychol. 85, 348–362. 10.1037/0022-3514.85.2.348 12916575

[B102] Gyurak A. Gross J. J. Etkin A. (2011). Explicit and implicit emotion regulation: a dual-process framework. Cogn. Emot. 25, 400–412. 10.1080/02699931.2010.544160 21432682 PMC3280343

[B23] Hiser J. Koenigs M. (2018). The multifaceted role of the ventromedial prefrontal cortex in emotion, decision making, social cognition, and psychopathology. Biol. Psychiatry 83, 638–647. 10.1016/j.biopsych.2017.10.030 29275839 PMC5862740

[B24] Hutchison R. M. Womelsdorf T. Allen E. A. Bandettini P. A. Calhoun V. D. Corbetta M.. (2013). Dynamic functional connectivity: promise, issues, and interpretations. Neuroimage 80, 360–378. 10.1016/j.neuroimage.2013.05.079 23707587 PMC3807588

[B25] Jacob Y. Winetraub Y. Raz G. Ben-Simon E. Okon-Singer H. Rosenberg-Katz K.. (2016). Dependency network analysis (DEPNA) reveals context related influence of brain network nodes. Sci. Rep. 6:27444. 10.1038/srep27444 27271458 PMC4895213

[B26] Johnson E. H. (1990). The Deadly Emotions: The Role of Anger, Hostility, and Aggression in Health and Emotional Well-Being. New York, NY: Praeger Publishers.

[B27] Kenett D. Y. Tumminello M. Madi A. Gur-Gershgoren G. Mantegna R. N. Ben-Jacob E. (2010). Dominating clasp of the financial sector revealed by partial correlation analysis of the stock market. PLoS One 5:e15032. 10.1371/journal.pone.0015032 21188140 PMC3004792

[B28] Kinnison J. Padmala S. Choi J.-M. Pessoa L. (2012). Network analysis reveals increased integration during emotional and motivational processing. J. Neurosci. 32, 8361–8372. 10.1523/JNEUROSCI.0821-12.2012 22699916 PMC3400262

[B29] Kober H. Barrett L. F. Joseph J. Bliss-Moreau E. Lindquist K. Wager T. D. (2008). Functional grouping and cortical-subcortical interactions in emotion: a meta-analysis of neuroimaging studies. Neuroimage 42, 998–1031. 10.1016/j.neuroimage.2008.03.059 18579414 PMC2752702

[B30] LeDoux J. E. (1996). The Emotional Brain. New York, NY: Simon & Schuster.

[B31] Levy D. J. Glimcher P. W. (2012). The root of all value: a neural common currency for choice. Curr. Opin. Neurobiol. 22, 1027–1038. 10.1016/j.conb.2012.06.001 22766486 PMC4093837

[B32] Lin T. Gilam G. Raz G. Or-Borichev A. Bar-Haim Y. Fruchter E.. (2017). Accessible neurobehavioral anger-related markers for vulnerability to post-traumatic stress symptoms in a population of male soldiers. Front. Behav. Neurosci. 11:38. 10.3389/fnbeh.2017.00038 28326027 PMC5339223

[B33] Lindquist K. A. Wager T. D. Kober H. Bliss-Moreau E. Barrett L. F. (2012). The brain basis of emotion: a meta-analytic review. Behav. Brain Sci. 35, 121–143. 10.1017/s0140525x11000446 22617651 PMC4329228

[B34] Mazzola V. Arciero G. Fazio L. Lanciano T. Gelao B. Popolizio T.. (2016). What impact does an angry context have upon us? The effect of anger on functional connectivity of the right insula and superior temporal gyri. Front. Behav. Neurosci. 10:109. 10.3389/fnbeh.2016.00109 27375449 PMC4893496

[B35] McDonald A. J. Mascagni F. Guo L. (1996). Projections of the medial and lateral prefrontal cortices to the amygdala: a *Phaseolus vulgaris* leucoagglutinin study in the rat. Neuroscience 71, 55–75. 10.1016/0306-4522(95)00417-3 8834392

[B36] Mitchell J. P. (2009). Social psychology as a natural kind. Trends Cogn. Sci. 13, 246–251. 10.1016/j.tics.2009.03.008 19427258 PMC2935896

[B37] Mograbi A. (2005). Avenge but one of my two eyes.

[B38] Morawetz C. Bode S. Derntl B. Heekeren H. R. (2017). The effect of strategies, goals and stimulus material on the neural mechanisms of emotion regulation: a meta-analysis of fMRI studies. Neurosci. Biobehav. Rev. 72, 111–128. 10.1016/j.neubiorev.2016.11.014 27894828

[B39] Ochsner K. N. Silvers J. A. Buhle J. T. (2012). Functional imaging studies of emotion regulation: a synthetic review and evolving model of the cognitive control of emotion. Ann. N Y Acad. Sci. 1251, E1–E24. 10.1111/j.1749-6632.2012.06751.x 23025352 PMC4133790

[B101] Phillips M. L. Ladouceur C. D. Drevets W. C. (2008). A neural model of voluntary and automatic emotion regulation: implications for understanding the pathophysiology and neurodevelopment of bipolar disorder. Mol. Psychiatry 13, 829–857. 10.1038/mp.2008.65 18574483 PMC2745893

[B40] Raz G. Hagin B. Hendler T. (2013). “E-motion pictures of the brain: recursive paths between affective neuroscience and film studies,” in Psychocinematics: Exploring Cognition at the Movies, ed. Shimamura P. (New York, NY: Oxford University Press), 285–313.

[B41] Raz G. Touroutoglou A. Wilson-Mendenhall C. Gilam G. Lin T. Gonen T.. (2016). Functional connectivity dynamics during film viewing reveal common networks for different emotional experiences. Cogn. Affect. Behav. Neurosci. 16, 709–723. 10.3758/s13415-016-0425-4 27142636

[B42] Raz G. Winetraub Y. Jacob Y. Kinreich S. Maron-Katz A. Shaham G.. (2012). Portraying emotions at their unfolding: a multilayered approach for probing dynamics of neural networks. Neuroimage 60, 1448–1461. 10.1016/j.neuroimage.2011.12.084 22285693

[B43] Rodgers J. L. Nicewander W. A. (1988). Thirteen ways to look at the correlation coefficient. Am. Stat. 42, 59–66. 10.2307/2685263

[B44] Rolls E. T. (2004). The functions of the orbitofrontal cortex. Brain Cogn. 55, 11–29. 10.1016/S0278-2626(03)00277-X 15134840

[B45] Rosell D. R. Siever L. J. (2015). The neurobiology of aggression and violence. CNS Spectr. 20, 254–279. 10.1017/S109285291500019X 25936249

[B46] Roy M. Shohamy D. Wager T. D. (2012). Ventromedial prefrontal-subcortical systems and the generation of affective meaning. Trends Cogn. Sci. 16, 147–156. 10.1016/j.tics.2012.01.005 22310704 PMC3318966

[B47] Seeley W. W. Menon V. Schatzberg A. F. Keller J. Glover G. H. Kenna H.. (2007). Dissociable intrinsic connectivity networks for salience processing and executive control. J. Neurosci. 27, 2349–2356. 10.1523/JNEUROSCI.5587-06.2007 17329432 PMC2680293

[B48] Shapira Y. Kenett D. Y. Ben-Jacob E. (2009). The Index cohesive effect on stock market correlations. Eur. Phys. J. B 72, 657–669. 10.1140/epjb/e2009-00384-y

[B49] Spielberger C. D. Sydeman S. J. Owen A. E. Marsh B. J. (1999). “Measuring anxiety and anger with the state-trait anxiety inventory (STAI) and the state-trait anger expression inventory (STAXI),” in The Use of Psychological Testing for Treatment Planning and Outcomes Assessment, 2nd Edn., ed. Maruish M. E. (New Jersey, NJ: Lawrence Erlbaum Associates), 993–1021.

[B103] Xia M. Wang J. He Y. (2013). BrainNet Viewer: a network visualization tool for human brain connectomics. PLoS One 8:e68910. 10.1371/journal.pone.0068910 23861951 PMC3701683

