# Supplementary material for: Anger Modulates Influence Hierarchies Within and Between Emotional Reactivity and Regulation Networks
Source: Front Behav Neurosci. 2018 Apr 4;12:60. doi: 10.3389/fnbeh.2018.00060 (PMC5897670; doi:10.3389/fnbeh.2018.00060)
Supplement: Supplementary file 1 [file Table_1.PDF]

Anger Modulates Influence Hierarchies Within and Between Emotional Reactivity and Regulation Networks

Supplemental Information

D_{EP}NA feature characteristics and interpretation

D_{EP}NA analysis (Jacob et al., 2016) provides a variety of measures ranging from a high resolution influence upon a single specific edge (i.e. correlation influence), to a broader region specific degree of influence (i.e. dependency matrix), a system level influence on an entire network (i.e. *'Influencing Degree'* and *'Influenced Degree'*), and up to a global level of influence of one network upon another (i.e. total inter-network influence). Each of these features can then be tested on different task conditions, groups, or in relation to individual behavioral measures. Table S1 represents our effort to summarize all D_{EP}NA measure characteristics and interpretations.

Table S1. Summary of DEPNA measures- details and characteristics

DEPNA measure	Definition	Characteristics	Interpretation
**Correlation influence** 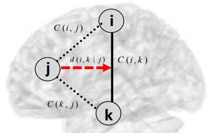 $d(i,k\mid j) = C(i,k) - PartialCorr(i,k\mid j)$	The influence of a specific region j signal on the correlation between a pair of different regions i and k signals. This measure is calculated according to the difference between the correlation C(i,k) and the partial correlation given j - PC(i,k|j).	• This quantity is large only when a significant fraction of the correlation between regions i and k signals can be explained in terms of region j.	• The correlation influence isa quantity of the effect a third node signal had over the correlation.<br>• The 'correlation influence' measure is not a measure of correlation (i.e. co-linearity between two signals).
**Dependency matrix** 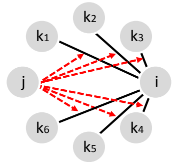 $D(i,j) = \frac{1}{N-1}\sum_{k \neq j}^{N-1} d(i,k\mid j)$	The dependency matrix D(i,j) element is the influence of region j on region i. This measure is calculated as the average correlation influence of region j on the correlations of region i with all other regions k in the network C(i,k). In order to avoid cases when we sum over elements of different signs we sum over positive influences only.	• The dependency matrix is nonsymmetrical directed matrix since the influence of region j on region i is not equal to the influence of region i on region j.	• The dependency matrix allows for a directed graph of the brain network.<br>• The dependency matrix measures do not infer causal influence in a true sense, rather infer the network' hierarchy of influence based on correlational influences.
**Influencing Degree** 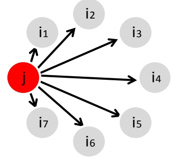 $Influencing\ Degree(j) = \sum_{i \neq j}^{N-1} D(i,j)$	The total influence of region j on the entire network. This measure is quantified as the sum of the influences D(i,j) of j on all other regions i.	• Provide the hierarchy of efferent (output) influence on the network.<br>• The higher the region's 'Influencing Degree' the more it influenced all other connections in the network.	• Regions with a high 'Influencing Degree' are more likely to generate the cognitive process.<br>• The longer the region processes the information (sustained activation), the higher it's influence on the entire network.
**Influenced Degree** 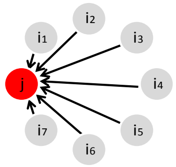 $Influenced\ Degree(j) = \sum_{i \neq j}^{N-1} D(j,i)$	The total influence of the entire network on the specific region j. This measure is quantified as the sum of all the influences D(j,i) of all regions i in the network on region j.	• Provide the hierarchy of afferent (input) influence by the network.<br>• The higher the region's 'Influenced Degree' the more it was dependent or influenced by all the other regions in the network.	• The further downstream the node is in the network, the higher its 'Influenced Degree', however, it was found to be very sensitive to the SNR.<br>• Regions with a high 'Influenced Degree' are more likely to be simultaneously influenced by many other regions.<br>• An increased 'Influenced Degree' pattern compared to baseline of the entire network indicates network's integration (i.e. all regions are more influenced by all other regions).<br>• A decreased 'Influenced Degree' pattern indicates network segregation.

Summary of DEPNA measures- details and characteristics (Continued)

DepNA measure	Definition	Characteristics	Interpretation
**Intra-network influence**<br>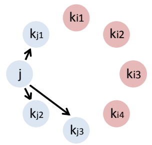<br>$Influencing\ Degree_{Intra}(j) = \sum_{kj}^{Nkj} D(kj, j)$	The total influence of region j on the entire sub-network to which it belongs. This measure is quantified as the sum of the influences D(kj,j) of region j on all other regions kj within the Kj network.	• Provide the hierarchy of afferent (input) influence within the sub-network.<br>• The higher the region's intra-network influence degree the more it influenced all other connections within its sub-network.	• Same as 'Influencing Degree' only within a smaller sub-network.
**Inter-network influence**<br>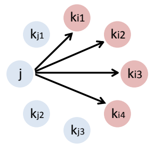<br>$Influencing\ Degree_{Inter}(j) = \sum_{i}^{Nki} D(ki, j)$	The total influence of region j on different sub-network regions. This measure is quantified as the sum of the influences D(ki,j) of region j on all other regions ki within the Ki network.	• Provide the hierarchy of efferent (output) influences of regions from one sub-network only on the connections of different sub-network regions.<br>• The higher the region's inter-networks influence degree the more it influenced all other connections within the other sub-network and between the two sub-networks.	• Regions with a high inter-network influence are more likely to integrate between the two networks.
**Total inter-networks influence**<br>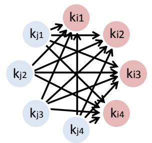<br>$Total\ Inter-Network(Kj) = \sum_{kj}^{Nkj} \sum_{ki}^{Nki} D(ki, kj)$	The total influence of sub-network Kj on sub-network Ki. This measure is quantified as the sum of all the influences D(ki,kj) of all kj regions within the Kj sub-network on all regions ki within the Ki sub-network.	• This quantity is large only when a significant fraction of the correlation between regions i and k signals can be explained in terms of region j.	• The 'correlation influence' measure is not a measure of correlation (i.e. co-linearity between two signals), rather a quantity of the effect a third node signal had over the correlation.

Spatial specificity results

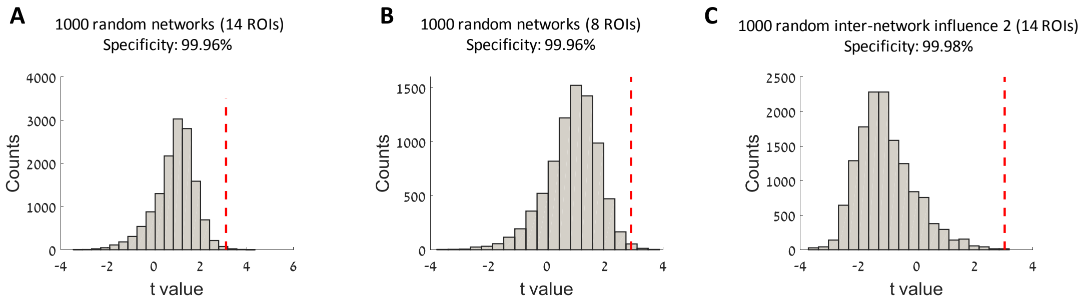

Figure S1: Specificity of the D_{EP}NA findings using bootstrapping analysis. Histograms of the paired t-test t statistic values for 1000 randomized networks generated by identical analyses of random sets of gray matter regions are presented. The red lines indicate the position of the results of the comparison of interest within the population of the corresponding values for randomized comparisons. **(A)** Spatial specificity test for the vmPFC correlational influence on the entire system configuration (t=3.11) consisted of 14 randomized ROIs. **(B)** Spatial specificity test for the vmPFC correlational influence on the regulation network configuration (t=2.90) consisted of 8 randomized ROIs. **(C)** Spatial specificity test for the vmPFC correlational influence on the connectivity between the reactivity and regulation networks configuration (t=3.10) consisted of 14 randomized ROIs. All tests were found to be statistically significant using the bootstrapping analyses.

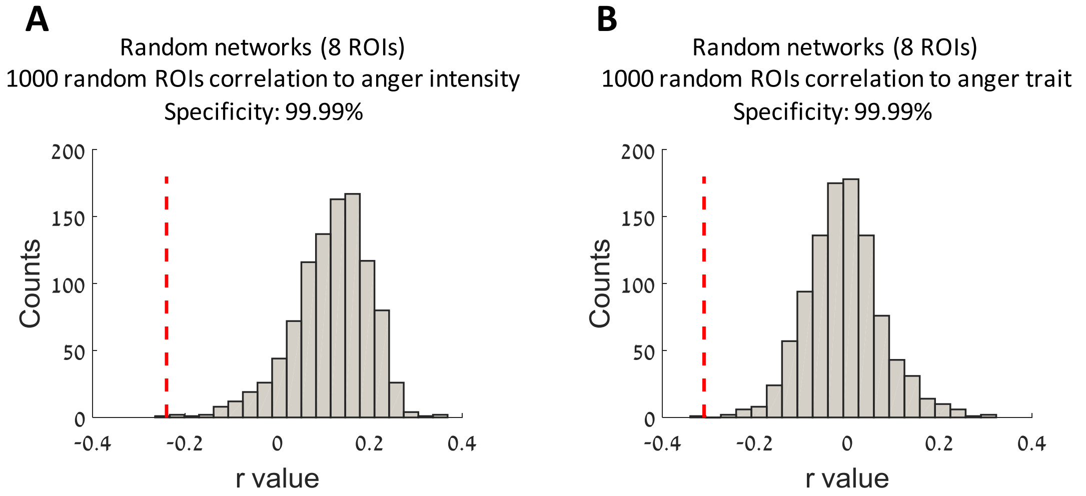

Figure S2: Specificity of the associations between the neural and behavioral indices. Histograms of Pearson correlation coefficients (r-values) for 1000 randomized ROIs DEPNA measures correlations to individual behavioral indices are presented. The red lines indicate the position of the observed results of the comparison of interest. **(A)** Specificity test for the vmPFC impact on the regulation network correlation to anger intensity (r=-0.24) and **(B)** anger trait (r=-0.31). All tests were found to be statistically significant using the bootstrapping analyses.

Controlling for order effect

As the dynamics of the film excerpt we used in this study are characterized by a gradual increase in anger intensity, resulting in a peak in this experience towards the end of the excerpt, differences between the two emotional anger states could be attributed to order effects. In order to address this issue analytically we divided the data into two low- and two high anger periods (two intervals of 22 TRs) and applied the D_{EP}NA analysis on each of these subsequent time frames. We then conducted a between-periods paired t-test for each region's *'Influencing Degree'* (total of 14 ROIs).

Considering a simple order effect, one would expect to obtain a linear increase in D_{EP}NA features as time progresses within each anger period (i.e., high or low). These analyses results do not support an order effect as there is no evidence for such a gradual increase or difference in *'Influencing Degree'* measures within each category as time progresses ($p > 0.06$., for all comparisons) (Figure S3).

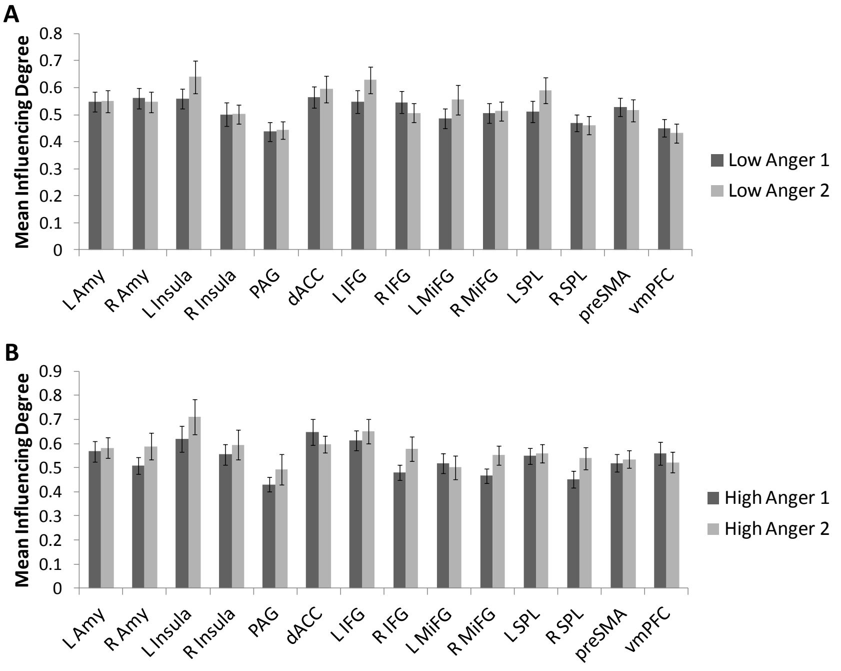

Figure S3: Order effect specificity of the D_{EP}NA findings. Each emotional time period was divided into two emotional subsequent time frames. DEPNA was conducted on each of these time periods and compared within each emotional category (low and high anger). The nodes' 'Influencing Degree' observed during the two low anger **(A)** and two high anger **(B)** periods averaged over all 74 subjects . As expected, none of these analyses were found to be significantly different between the two time periods during the low- and high-anger time frames.

References

Jacob Y., Winetraub Y., Raz G., Ben-Simon E., Okon-Singer H., Rosenberg-Katz K., Hendler T., Ben-Jacob E. (2016) Dependency Network Analysis (DEPNA) Reveals Context Related Influence of Brain Network Nodes. Sci Rep 6:27444. DOI: srep27444 [pii]

10.1038/srep27444 [doi].
